# Decreased Peripheral Naïve T Cell Number and Its Role in Predicting Cardiovascular and Infection Events in Hemodialysis Patients

**DOI:** 10.3389/fimmu.2021.644627

**Published:** 2021-03-17

**Authors:** Fangfang Xiang, Xuesen Cao, Xiaohong Chen, Zhen Zhang, Xiaoqiang Ding, Jianzhou Zou, Bo Shen

**Affiliations:** ^1^Department of Nephrology, Zhongshan Hospital, Fudan University, Shanghai, China; ^2^Shanghai Key Laboratory of Renal Disease and Blood Purification, Shanghai, China; ^3^Shanghai Medical Center of Kidney, Shanghai, China; ^4^Shanghai Institute of Kidney and Dialysis, Shanghai, China

**Keywords:** hemodialysis, T-cell senescence, naïve T cells, cardiovascular event, infection

## Abstract

Patients with end-stage renal disease (ESRD) are at high risk of morbidity and mortality from cardiovascular and infectious diseases, which have been found to be associated with a disturbed immune response. Accelerated T-cell senescence is prevalent in these patients and considered a significant factor contributing to increased risk of various morbidities. Nevertheless, few studies have explicated the relevance of T-cell senescence to these fatal morbidities in ESRD patients. In this study, we designed a longitudinal prospective study to evaluate the influence of T-cell senescence on cardiovascular events (CVEs) and infections in hemodialysis (HD) patients. Clinical outcomes of 404 patients who had been on HD treatment for at least 6 months were evaluated with respect to T-cell senescence determined using flow cytometry. We found that T-cell senescence was associated with systemic inflammation. High-sensitivity C-reactive protein was positively associated with decreased naïve T cell levels. Elevated tumor necrosis factor-α and interleukin 6 levels were significantly associated with lower central memory T cell and higher T effector memory CD45RA cell levels. Decreased CD4^+^ naïve T cell count was independently associated with CVEs, whereas decreased CD8^+^ naïve T cell count was independently associated with infection episodes in HD patients. In conclusion, HD patients exhibited accelerated T-cell senescence, which was positively related to inflammation. A reduction of naïve T cell could be a strong predictor of CVEs and infection episodes in HD patients.

## Introduction

End-stage renal disease (ESRD), considered as a public health concern, affects more than 1.5 million people worldwide (1). Patients with ESRD usually have a high risk of life-threatening comorbidities, especially cardiovascular and infectious diseases. According to the U.S. Renal Data System, ESRD patients have a 25% annual mortality rate, and almost 50% patient deaths are attributed to cardiovascular complications (2). Infection is the second leading cause of death, accounting for 35% of all-cause mortality (3). It has been proposed that chronic kidney disease may be a model of premature aging, since uremia could induce premature senescence and many aging-related complications are prevalent in ESRD patients, including those with cardiovascular diseases (CVDs) and infections (4). Recent evidence suggests that uremia can induce T-cell senescence, indicated by a lower thymic output of naïve T cells, a decline in T-cell telomere length, and an increase in differentiation toward the terminal differentiated memory phenotype; T-cell senescence is more pronounced in patients undergoing hemodialysis (HD) therapy (5, 6). Compared with physiological aging, ESRD seems to have the ability to increase the immunological age of T cells by 20–30 years (7). In terms of function, T cells in ESRD patients are pre-activated by secreting more inflammatory cytokines in the resting state, leading to persistent inflammation and providing a breeding ground for CVD (8, 9). On the contrary, T cells in ESRD patients have diminished reaction toward pathogen stimulation, with susceptibility to apoptotic death after activation (9), reduced humoral response to vaccination (10), and impaired maintenance of specific T cell memory (11), resulting in a high incidence of infection. Hence, interventions targeting T cell function could improve morbidity and mortality in such patients.

While it is well-recognized that ESRD-related T cell dysfunction is prominent, few studies have explicated the relevance of T-cell senescence to the fatal morbidity resulting from ESRD, and existing results are based on different markers of immune senescence. It has been reported that telomere length shortening is associated with a higher risk of death, reduced thymic output is associated with severe infection episodes, and terminally differentiated CD8^+^T cell expansion is closely linked to accelerated atherosclerosis in ESRD patients (5). CD4^+^CD28^-^T cells, as a terminal differentiated memory phenotype, were independently associated with the presence of atherosclerotic disease in ESRD patients (12). Cytomegalovirus (CMV) infection is considered to act as a critical factor for accelerated T-cell senescence in ESRD patients by exacerbating the selective depletion of naïve T cells and clonal expansion of memory T cells (13). However, since most patients with ESRD are CMV-seropositive (14, 15), it is difficult to distinguish the CMV-independent effects of T-cell aging in ESRD. The question that then arises is whether it would be possible to find one consistent marker for evaluating overall immunological age, assessing the risk of multiple complications, and aiding early intervention in ESRD patients.

Depletion of naïve T cells, the most significant and consistent change reported during aging, is also prevalent in ESRD (8, 15). Our previous study findings revealed that a decrease in the number of naïve T cells is significantly associated with increased mortality in HD patients (16), supporting the idea that selective reduction of naïve T cell is a critical feature in this population and may impact clinical outcomes profoundly. In the present study, we prospectively analyzed whether T-cell senescence is associated with cardiovascular events (CVEs) and infectious episodes in HD patients and aimed to find valuable markers for clinically evaluating immunological aging and predicting risk of ESRD.

## Materials and Methods

### Study Population

This current study included patients who had been on HD treatment for at least 6 months in the Blood Purification Center, Department of Nephrology, Zhongshan Hospital, Fudan University. Patients were enrolled from August to September, 2016 and followed weekly. Individuals who experienced CVE or infection within 3 months were excluded. Those with evidence of hematological diseases, rheumatic diseases, active malignancies, and history of human immunodeficiency virus infection or using immunosuppressants were also excluded. Follow-up lasted for 2 years and ended in October 2018. During follow-up, CVEs and infection episodes were documented. CVEs were defined as coronary artery disease, congestive heart failure, stroke, and peripheral arterial occlusive disease. Infection episodes were defined as infectious diseases requiring regular intravenous antibiotics in hospital or emergency department.

We obtained blood samples from the arterial site of vascular access before the start of the HD session in the middle of the week. Anti-CMV-IgM and IgG antibodies were detected using the Roche Elecsys assay. All procedures were performed at the Department of Clinical Chemistry, Zhongshan Hospital, Fudan University using standard methods. Written informed consent was obtained from all patients that met the inclusion criteria. This study was approved by the Ethics committee of Zhongshan Hospital, Fudan University.

### Cell Preparation and Flow Cytometry Analysis

On the day of blood drawing, blood samples mixed with heparin anticoagulant were lysed with red blood cell lysis solution and 0.1 mM EDTA and prepared for flow cytometry analysis with the following fluorescein-conjugated monoclonal antibodies: CD3-PE (Bio- Legend, San Diego, CA, USA), CD4-APC (eBioscience, San Diego, CA, USA), CD8a-Percp/Cy5.5 (eBioscience), CD45RO-FITC (Miltenyi Biotec, Bergisch Gladbach, Germany), and CCR7-APC/Cy7 (BioLegend). The relative expression of CD45RO and CCR7 was used to identify naïve T cell (T_Naïve_, CD45RO^−^ CCR7^+^), central memory T cell (T_CM_, CD45RO^+^ CCR7^+^), effector memory T cell (T_EM_, CD45RO^+^ CCR7^−^), and T effector memory CD45RA cell (T_EMRA_, CD45RO^−^ CCR7^−^) subsets of CD4^+^ or CD8^+^ T cells. These markers were selected according to previous studies (7, 17). The immunophenotyping methods and gating strategy have been elaborated in the supplementary materials (**Figure S1**).

### Statistical Analysis

All data are expressed as mean ± standard deviation or median (interquartile range), as appropriate. Correlations between T cell parameters and laboratory variables were tested using a non-parametric Spearman rank analysis. Free survival of CVEs and infection episodes were estimated using the Kaplan–Meier curve, and differences between groups were examined using the log-rank test. Univariate Cox regression analysis was used to identify predictors of CVE and infection. Significant predictors were subsequently added to the multivariable model, and backward stepwise Cox regression identified the most parsimonious model. The probability used for the stepwise regression was set at 0.05 for entry of variables and 0.1 for removal of variables. The results of the Cox proportional hazards analysis are presented as the hazard ratio (HR) and 95% confidence interval (95% CI). Statistical significance was considered at *P* < 0.05. All statistical analyses were performed using SPSS version 20.0.

## Results

### Demographic and Clinical Characteristics of Patients

A total of 404 patients (248 men and 156 women) were enrolled in this study. The average age of patients was 59.4 ± 14.6 years. The median time in HD was 53 (26, 80) months. Of the 404 patients, 94 (23.3%) had diabetes mellitus and 324 (80.2%) had hypertension. The overall frequency of CVD in this cohort was 30.7%; stroke and congestive heart failure were the most prevalent complications, followed by coronary artery disease and peripheral arterial occlusive disease. The underlying kidney diseases included chronic glomerulonephritis (46.8%), diabetic nephropathy (16.8%), polycystic kidney disease (9.4%), hypertension renal disease (3.5%), others (10.9%), and unknown (12.6%). Only one patient (0.2%) was seropositive for CMV-IgM, and 401 patients (99.3%) were seropositive for CMV-IgG. The median level of CMV-IgG was 468 U/ml, and 189 patients (46.8%) had CMV-IgG titers exceeding the upper limit of 500 U/ml. **Table 1** presents the baseline characteristics of the study population.

**Table 1:** Demographic data of the study population.

Variable	mean ± SD/median (interquartile range)
Age, years	59.4 ± 14.6
Time on HD, months	53 (26,80)
Male (%)	248 (61.4%)
Diabetes mellitus (%)	94 (23.3%)
CVD history (%)	124 (30.7%)
Hypertension (%)	324 (80.2%)
CMV seropositive (%)	401 (99.3%)
BMI (kg/m^2^)	21.5 ± 3.2
Kt/Vurea	1.31 ± 0.57
Hemoglobin, g/L	112.4 ± 15.9
White blood cell, ×10^9^/L	6.56 ± 2.02
Lymphocytes, ×10^9^/L	1.3 ± 0.5
Albumin, g/L	39.0 ± 3.2
Prealbumin, g/L	0.32 ± 0.13
Creatinine, μmol/L	1,000.3 ± 277.3
Uric acid, mmol/L	441.6 ± 88.8
Calcium, mmol/L	2.32 ± 0.24
Phosphorus, mmol/L	2.17 ± 0.65
Total cholesterol, mmol/L	4.11 ± 1.07
Triglyceride, mmol/L	1.45 (1.03, 2.23)
LDL-C, mmol/L	2.27 ± 0.87
HDL-C, mmol/L	1.06 ± 0.59
Homocysteine, μmol/L	34.7 (26.4, 46.6)
NT-proBNP, pg/ml	3,882.0 (1,782.3, 10,324.2)
iPTH, pg/ml	260.7 (150.3, 407.2)
Ferritin, pg/ml	296.9 (139.3, 495.5)
hsCRP, mg/L	4.0 (1.4, 10.2)
TNF-α, pg/ml	33.4 (22.8, 58.8)
IL-6, pg/ml	9.6 (4.2, 36.2)

CVD, cardiovascular disease; CMV, cytomegalovirus; BMI, Body mass index; LDL-C, low density lipoprotein–cholesterol; HDL-C, high density lipoprotein–cholesterol; NT-proBNP, N-terminal pro-brain natriuretic peptide; iPTH, intact parathyroid hormone; hsCRP, high-sensitivity C-reactive protein; TNF-α, tumor necrosis factor-α; IL-6, interleukin 6.

### T-Cell Senescence Is Associated With Systemic Inflammation in HD Patients

We examined the association between T cell subsets and circulating inflammatory markers at enrollment. As shown in **Table 2**, high-sensitivity C-reactive protein (hsCRP) was positively associated with decreased T_Naïve_ cell count in both CD4^+^ and CD8^+^ T cell compartments (*p* < 0.05). Meanwhile, elevated tumor necrosis factor-*α* (TNF-*α*) and interleukin 6 (IL-6) levels were significantly associated with lower CD4^+^ T_CM_ and higher CD4^+^ T_EMRA_ levels (*p* < 0.001).

**Table 2:** Correlations between T cell subsets and inflammatory markers in hemodialysis patients.

	TNF-α		IL-6		hsCRP	
	Correlation coefficient	*p*	Correlation coefficient	*p*	Correlation coefficient	*p*
**Cell subset percentage**						
CD4^+^T cells %	−0.198^**^	<0.001	−0.093	0.062	0.005	NS
CD4^+^ T_Naïve_%	0.015	NS	0.026	NS	−0.097	0.052
CD4^+^T_CM_%	−.225^**^	<0.001	−.248^**^	<0.001	0.071	NS
CD4^+^T_EM_%	−0.032	NS	−0.016	NS	0.096	0.054
CD4^+^T_EMRA_%	0.321^**^	<0.001	0.312^**^	<0.001	−0.034	NS
CD8^+^T cells%	0.180^**^	<0.001	0.05	NS	−0.002	NS
CD8^+^ T_Naïve_%	−0.016	NS	−0.023	NS	−0.146^*^	0.003
CD8^+^ T_CM_%	−0.083	0.095	−0.046	NS	0.052	NS
CD8 ^+^T_EM_%	−0.121^*^	0.015	−0.061	NS	0.100^*^	0.045
CD8^+^T_EMRA_%	0.104^*^	0.037	0.073	NS	0.073	NS
**Absolute cell number**						
CD4^+^T cells (cells/μl)	−0.085	0.089	−0.129^*^	001	−0.062	NS
CD4^+^ T_Naïve_ (cells/μl)	−0.058	NS	−0.085	0.087	−0.108^*^	0.03
CD4^+^T_CM_ (cells/μl)	−0.215^*^	0.001	−0.260^*^	0.001	0.012	NS
CD4^+^T_EM_ (cells/μl)	−0.021	NS	−0.075	NS	0.043	NS
CD4^+^T_EMRA_ (cells/μl)	0.242^*^	0.001	0.205^*^	0.001	−0.063	NS
CD8^+^T cells (cells/μl)	0.075	NS	−0.059	NS	−0.035	NS
CD8^+^ T_Naïve_ (cells/μl)	0.033	0.051	−0.062	NS	−0.148^*^	0.003
CD8^+^ T_CM_ (cells/μl)	−0.07	NS	−0.084	0.093	0.013	NS
CD8 ^+^T_EM_ (cells/μl)	−0.033	NS	−0.103^*^	0.039	0.044	NS
CD8^+^T_EMRA_ (cells/μl)	−0.016	NS	0.044	NS	0.046	NS

Spearman rank analysis was applied to investigate the relationship between inflammatory markers and T cell subset level, including the percentages as well as absolute cell counts of naïve (T_Naïve_), central memory (T_CM_), effector memory (T_EM_), and effector memory CD45RA (T_EMRA_) subsets. *p value < 0.05. **p value < 0.001. NS, non-significant, p value > 0.1.

### Decreased CD4^+^ T_Naïve_ Cell Count as a Predictor of CVEs in HD Patients

**Figure 1:**
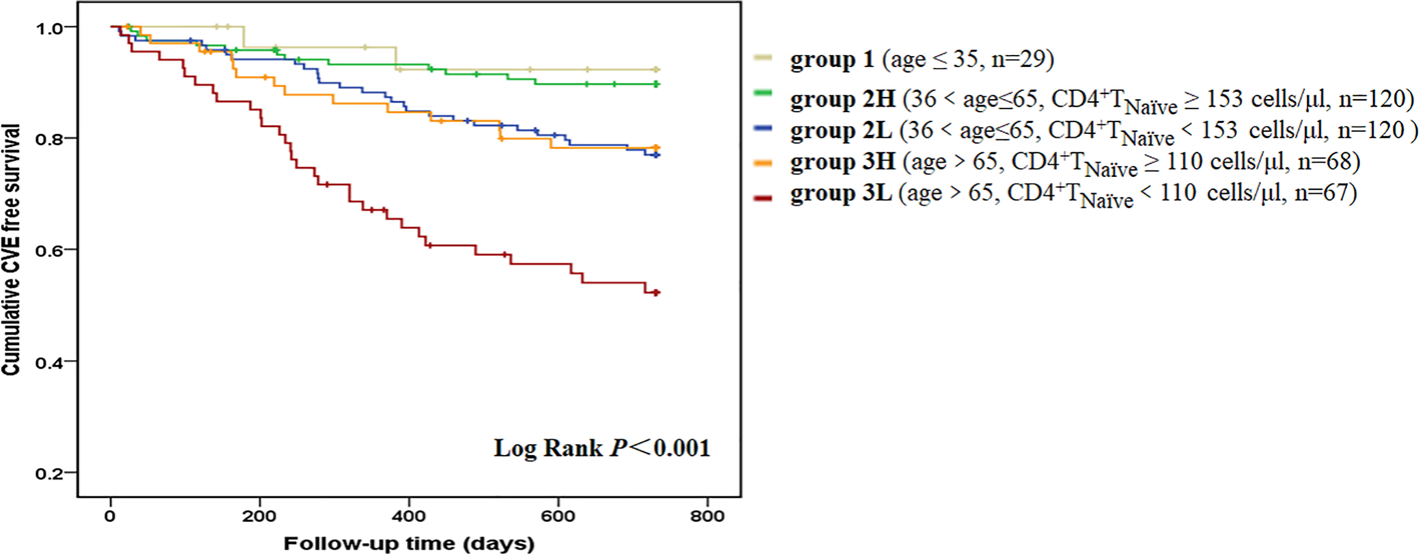
CVE-free survival curves according to age-CD4^+^ T_Naïve_ group. We divided the patients into five groups according to age and CD4^+^ T_Naïve_ cell count. Group 1 included young patients (age ≤35 years old, n = 29). Group 2L included middle-aged patients with a lower CD4^+^ T_Naïve_ cell count [36 < age (years) ≤ 65, CD4^+^ T_Naïve_ < 153 cells/μl, n = 120]. Group 2H included middle-aged patients with a higher CD4^+^ T_Naïve_ cell count [36 < age (years) ≤ 65, CD4^+^T_Naïve_ ≥ 153 cells/μl, n = 120]. Group 3L included old patients with a lower CD4^+^ T_Naïve_ cell count (age > 65 years old, CD4^+^T_Naïve_ < 110 cells/μl, n = 67). Group 3H included old patients with a higher CD4^+^ T_Naïve_ cell count (age > 65 years old, CD4^+^ T_Naïve_ ≥ 110 cells/μl, n = 68). Kaplan-Meier analysis revealed that survival rate was significantly different among the five age-CD4^+^ T_Naïve_ groups (*p* < 0.001). In pairwise comparison, patients with a lower CD4^+^ T_Naïve_ count had a significantly higher CVE incidence in both the middle-aged (*p* = 0.014) and old groups (*p* = 0.003). There was no difference between middle-aged patients with a lower CD4^+^ T_Naïve_ count and old patients with a higher CD4^+^ T_Naïve_ count.

During the 650 ± 176 days of follow-up, 86 patients (21.3%) experienced at least one CVE and a total of 99 CVEs were recorded. The incidence of CVE was 13.4% per year. A total of 42 patients died of CVEs, accounting for 56.8% of all-cause mortality. Furthermore, 32 patients had stroke and 14 died of it; 24 patients developed acute coronary syndrome and 12 died of it; 22 patients experienced at least one event of heart failure and 8 died of it; 12 patients developed lower extremity atherosclerotic occlusive disease and 4 died of it; and 4 patients died of sudden cardiac death. The median value of each T cell parameter was used in analyzing the correlation between CVEs. A lower absolute number/percentage of CD4^+^ T_Naïve_ as well as a higher percentage of CD4^+^ T_EM_ and CD8^+^ T_EM_ could significantly predict CVEs (**Figure S2**). When taking age into consideration, only CD4^+^ T_Naïve_ cells were shown to significantly predict CVEs. In the pairwise comparison, patients with a lower CD4^+^ T_Naïve_ count had a significantly higher CVE incidence in both the middle-aged [36 < age (years) ≤ 65, *p* = 0.014] and old (age > 65 years old, *p* = 0.003) groups. There was no difference in CVE incidence between middle-aged patients with a lower CD4^+^ T_Naïve_ count and old patients with a higher CD4^+^ T_Naïve_ count (**Figure 1**). In the univariate Cox proportional hazard model, other CVE predictors included older age, history of CVD and diabetes mellitus, usage of central venous catheter, lower serum levels of albumin, prealbumin, creatinine, and uric acid, and increased levels of white blood cell count, hsCRP, and N-terminal pro-brain natriuretic peptide (NT-proBNP) (**Table 3**). In the multivariate Cox hazard model, a decreased count of CD4^+^ T_Naïve_ cells along with older age, history of diabetes, history of CVD, as well as elevated white blood cell count and NT-proBNP was independently associated with CVEs (HR 0.430, 95% CI 0.253–0.731, *p* = 0.002).

**Table 3:** Cox hazard model for CVEs in hemodialysis patients.

Variables	Univariate Cox hazard model		Multivariate Cox hazard model^1^	
	HR (95% CI)	*P* value	HR (95% CI)	*P* value
Age (≥65 years old = 1)	2.967 (1.929, 4.564)	<0.001	1.747 (1.068, 2.857)	0.026
Sex (male = 1)	1.263 (0.810, 1.968)	0.303		
Diabetes mellitus (yes = 1)	2.767 (1.802, 4.249)	<0.001	1.687 (1.060, 2.683)	0.027
CVD (yes = 1)	5.169 (3.323, 8.034)	<0.001	3.118 (1.839, 5.286)	<0.001
Central venous catheter (yes = 1)	2.137(1.383, 3.302)	<0.001		
BMI (kg/m^2^)	0.961 (0.902, 1.024)	0.275		
Kt/Vurea	0.556 (0.288, 1.074)	0.081		
Time on HD (month)	0.996 (0.991, 1.001)	0.108		
CMV IgG (U/ml)^2^	1.003 (1.000,1.007)	0.084		
Hemoglobin (g/L)	0.989 (0.976, 1.003)	0.116		
White blood cell (×10^9^/L)	1.111 (1.011, 1.220)	0.029	1.155 (1.040, 1.283)	0.007
Albumin (g/L)	0.859 (0.804, 0.916)	<0.001		
Prealbumin (g/L)	0.023 (0.003, 0.199)	0.001		
Creatinine(μmol/L)	0.998 (0.998, 0.999)	<0.001		
Uric acid (mmol/L)	0.995 (0.993, 0.998)	<0.001		
Triglyceride (mmol/L)	0.804 (0.649, 0.996)	0.045		
LDL-C (mmol/L)	0.974 (0.758, 1.251)	0.835		
Phosphorus (mmol/L)	0.928 (0.664, 1.295)	0.660		
Calcium (mmol/L)	0.723 (0.297, 1.759)	0.475		
Log-iPTH (pg/ml)	1.107 (0.616, 1.989)	0.735		
β2-Microglobulin (mg/L)	1.012 (0.986, 1.040)	0.371		
Homocysteine (μmol/L)	1.001 (0.995, 1.007)	0.705		
Log-hsCRP (mg/L)	2.058 (1.439, 2.943)	<0.001		
Log-NT-proBNP (pg/ml)	4.407 (2.777, 6.994)	<0.001	2.388 (1.409, 4.048)	0.001
CD4^+^ T_Naïve_ count (≥137 cells/μl = 1)	0.352 (0.219, 0.553)	<0.001	0.430 (0.253, 0.731)	0.002
CD4^+^ T_Naïve_ % (≥36.7 = 1)	0.505 (0.325, 0.784)	0.002		
CD4^+^T_EM_% (≥33.2 = 1)	1.987 (1.279, 3.087)	0.002		
CD8^+^T_EM_% (≥22.0 = 1)	1.770 (1.143, 2.741)	0.011		

CVD, cardiovascular disease; BMI, Body mass index; HD, hemodialysis; CMV IgG, cytomegalovirus immunoglobulin G; LDL-C, low density lipoprotein–cholesterol; Log-iPTH, log transformed intact parathyroid hormone; Log-hsCRP, log transformed high-sensitivity C-reactive protein; Log-NT-proBNP, log transformed N-terminal pro-brain natriuretic peptide. T_Naïve_, naïve T cell; T_EM_, effector memory T cell.

^1^Backward conditional method was used. Model included each T cell parameters and was adjusted for age, gender, history of CVD, history of diabetes, types of vascular access, Kt/Vurea, CMV IgG, albumin, prealbumin, white blood cell, creatinine, uric acid, triglyceride, LDL-C, NT-proBNP, and hsCRP.

^2^For those with CMV-IgG titers exceeding the upper limit of 500 U/ml, the numbers were regarded as 500 U/ml.

### Decreased CD8^+^ T_Naïve_ Cell Count as a Predictor of Infection Episodes in HD Patients

**Figure 2:**
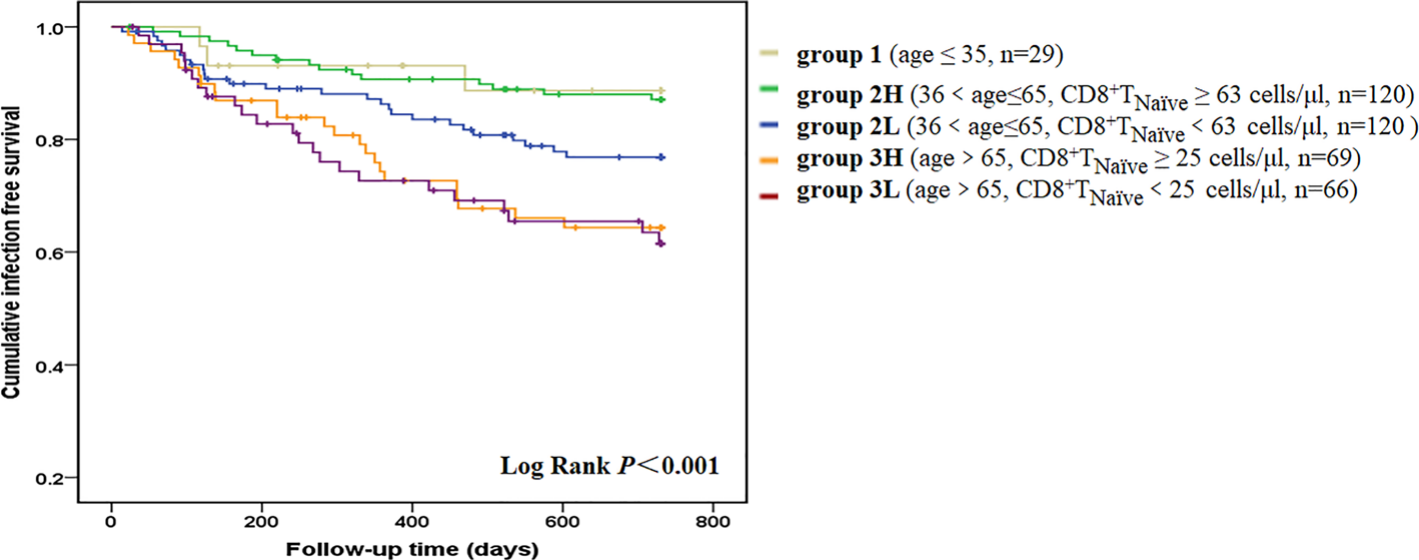
Infection-free survival curves according to age-CD8^+^ T_Naïve_ group. We divided the patients into five groups according to age and CD8^+^ T_Naïve_ cell count. Group 1 included young patients (age ≤35 years old, n = 29). Group 2L included middle-aged patients with a lower CD8^+^ T_Naïve_ cell count [36 < age (years) ≤ 65, CD8^+^ T_Naïve_ < 63 cells/μl, n = 120]. Group 2H included middle-aged patients with a higher CD8^+^ T_Naïve_ cell count [36 < age (years) ≤ 65, CD8^+^ T_Naïve_ ≥ 63 cells/μl, n = 120]. Group 3L included old patients with a lower CD8^+^ T_Naïve_ cell count (age > 65 years old, CD8^+^ T_Naïve_ < 25 cells/μl, n = 66]. Group 3H included old patients with a higher CD8^+^ T_Naïve_ cell count (age > 65 years old, CD8^+^ T_Naïve_ ≥ 25 cells/μl, n = 69). Kaplan-Meier analysis revealed that survival rate was significantly different among the five age-CD8^+^ T_Naïve_ groups (*p* < 0.001). In pairwise comparison, old patients had a significantly higher infection incidence, regardless of the CD8^+^ T_Naïve_ count. Patients with a lower CD8^+^ T_Naïve_ count in the middle-aged group had a significantly higher infection incidence than those with a higher CD8^+^ T_Naïve_ count in the same age group (*p* = 0.04).

A total of 90 patients (22.3%) experienced at least one infectious episode and 16 died of it, which accounted for 21.6% of all-cause mortality. The incidence of infection was 15.6% per year. A total of 97 infectious events were recorded. The following infections were reported: pulmonary infections [n = 55 (56.7%)], dialysis access-related infections [n = 14 (14.4%)], skin or joint infections [n = 9 (9.3%)], urinary or abdominal infections [n = 10 (10.3%)], septic shock [n = 3 (3.1%)], and infections at other sites or undocumented sites [n = 6 (6.2%)]. The median value of each T cell parameter was used for analyzing the correlation between infections. Decreased absolute count/percentage of CD8^+^ T_Naïve_ and increased percentage of CD8^+^ T_EMRA_ cells were significant predictors of infection (**Figure S3**). Although aging contributes to both infection and depletion of CD8^+^ T_Naïve_ cells, patients with a lower CD8^+^T_Naïve_ count in the middle-aged group [36 < age (years) ≤ 65] had a significantly higher infection incidence than those with a higher CD8^+^ T_Naïve_ count in the same age group (*p* = 0.04) (**Figure 2**). Other infection event predictors included a history of CVD, usage of central venous catheter, decreased levels of hemoglobin, albumin, prealbumin, creatinine, and uric acid, and increased serum levels of hsCRP, NT-proBNP, ferritin, and globulin (**Table 4**). In the multivariate Cox hazard model, a decreased count of CD8^+^ T_Naïve_ cells was independently associated with infection episodes in HD patients (HR 0.460, 95% CI 0.279–0.758, *p* = 0.002).

**Table 4:** Cox hazard model for infection incident in hemodialysis patients.

Variables	Univariate Cox hazard model		Multivariate Cox hazard model^1^	
	HR (95% CI)	*P* value	HR (95% CI)	*P* value
Age (≥65 years old = 1)	2.514 (1.658, 3.813)	<0.001		
Sex (male = 1)	1.207 (0.784, 1.858)	0.394		
Diabetes mellitus (yes = 1)	1.213 (0.755, 1.949)	0.425		
CVD (yes = 1)	1.724 (1.130, 2.630)	0.011		
Central venous catheter (yes = 1)	2.653(1.744, 4.036)	<0.001	2.225 (1.416, 3.497)	0.001
BMI (kg/m^2^)	0.985 (0.925, 1.048)	0.626		
Kt/Vurea	0.777 (0.481, 1.255)	0.303		
Time on HD (month)	0.999 (0.995, 1.003)	0.700		
Hemoglobin (g/L)	0.978 (0.967, 0.990)	<0.001	0.983 (0.970, 0.997)	0.014
White blood cell (×10^9^/L)	1.013 (0.915, 1.122)	0.800		
Albumin (g/L)	0.864 (0.812, 0.920)	<0.001		
Globulin (g/L)	1.048 (1.003, 1.095)	0.036	1.039 (0.996, 1.084)	0.074
Prealbumin (g/L)	0.079 (0.011, 0.540)	0.010		
Creatinine(μmol/L)	0.998 (0.998, 0.999)	<0.001		
Uric acid (mmol/L)	0.997 (0.994, 0.999)	0.007		
Phosphorus (mmol/L)	0.794 (0.571, 1.104)	0.171		
Calcium (mmol/L)	1.136 (0.475, 2.716)	0.775		
Log-iPTH (pg/ml)	0.902 (0.517, 1.573)	0.717		
Log-hsCRP (mg/L)	1.780 (1.268, 2.498)	<0.001		
Log-NT-proBNP (pg/ml)	2.180 (1.403, 3.388)	0.001	1.559 (0.977, 2.488)	0.062
Log-ferritin (pg/ml)	1.729 (1.002, 2.983)	0.049		
CD8^+^ T_Naïve_ count (≥46 cells/μl = 1)	0.469 (0.304, 0.725)	<0.001	0.460 (0.279, 0.758)	0.002
CD8^+^ T_Naïve_ % (≥19.7 = 1)	0.420 (0.270, 0.654)	<0.001		
CD8^+^T_EMRA_% (≥50.3 = 1)	1.902 (1.239, 2.920)	0.003	1.549 (0.978, 2.453)	0.062

CVD, cardiovascular disease; BMI, Body mass index; HD, hemodialysis; Log-iPTH, log transformed intact parathyroid hormone; Log-hsCRP, log transformed high-sensitivity C-reactive protein; Log-NT-proBNP, log transformed N-terminal pro-brain natriuretic peptide; T_Naïve_, naïve T cell; T_EMRA_, T effector memory CD45RA cells.

^1^Backward conditional method was used. Model included each T cell parameters and was adjusted for age, history of CVD, history of diabetes, types of vascular access, hemoglobin, albumin, prealbumin, creatinine, uric acid, globulin, ferritin, NT-proBNP, and hsCRP.

## Discussion

In the current study, CVEs and infections were the major complications accounting for more than 70% of all-cause mortality. Our study finding indicates that a decreased level of CD4^+^ naïve T cells is a strong predictor of CVEs, while a decreased level of CD8^+^ naïve T cells is a strong predictor of infectious episodes in HD patients. Loss of naïve T cells might be a hallmark of immune disturbance, leading to a more intense immune incompetence with profound clinical outcomes.

In the original model of the T cell system, naïve T cells are activated in the presence of infection, which then proliferate and generate heterogeneous classes of effector and memory cells with distinctive surface phenotypes, cytokine production abilities, and homing potentials (18). The T cell system has unique mechanisms of replenishment. Thymic T cell generation is the only way to add novel naïve T cells and enrich diversity; however, thymic function rapidly declines during adolescence and early adulthood and is quantitatively irrelevant throughout adult life (19). Instead, homeostatic proliferation is responsible for maintaining the size of the naïve T cell compartment and sustaining the richness of the T cell receptor repertoire (20). Generally, homeostatic proliferation in humans is efficient in maintaining a sizable CD4^+^ naïve T cell pool (21). CD8^+^ naïve T cells, on the contrary, are progressively lost with age, which induces a higher homeostatic proliferation of aged-CD8^+^ naïve T cells than that of aged-CD4^+^ naïve T cells (20).

To the best of our knowledge, this is the first study to identify a decrease in CD4^+^ naïve T cells as a novel CVE risk factor and a decrease in CD8^+^ naïve T cells as a novel infection risk factor in patients with ESRD. Notably, compelling data suggest profound lymphopenia of naïve T cells in both the CD4^+^ and CD8^+^ compartments in ESRD (15, 22), although the underlying mechanism is not sufficiently understood. It is evident from the literature that there is a reduced thymic output in ESRD (15, 22); however, the more important reason seems to be the failure to maintain quiescence in these cell compartments. Maintenance of quiescence is vital for naïve T cells to retain their self-renewal potential and differentiation plasticity throughout life. In circumstances of inflammation, T cells can leave their usual quiescent state and accumulate as partially differentiated cells, even in the absence of antigen stimulation (23, 24). In ESRD, inflammation is significantly enhanced with uremia (25), and dialysis treatment certainly exposes these patients to microbial products and other antigenic stimulations, which can lead to accelerated activation and turnover of naïve T cells. Thus, chronic inflammation could be responsible for the decreased naïve T cells in ESRD patients, which is supported by our finding that decreased levels of naïve T cells were correlated with elevated levels of the inflammation marker hsCPR in both CD4^+^ and CD8^+^ compartments. In earlier studies on aging, the decline in naïve T cells and relative expansion of memory and effector T cell populations were entirely due to chronic CMV stimulation (26). In this context, chronic immune stimulation could be the reason for accelerated T cell aging in ESRD patients, including at least the prevalent CMV infection, renal damage, uremia toxin retention, and increased reactive oxygen species generation. Any attempts to maintain the naïve T cell pool eventually lead to its further depletion and extinction, as such attempts result in the partial loss of stemness and incomplete differentiation and activation of negative regulatory programs (20, 27). In this context, decreased naïve T cells could represent their maladaptive behavior in ageing and even trigger a vicious cycle of aggravated immunosenescence. This is more so in case of CD4^+^ naïve T cells, as their shrinkage is not common during normal aging. Besides chronic kidney diseases, rheumatoid arthritis is another pathological condition wherein there are several lines of evidence of premature aging of T cells, indicating a defective DNA repair mechanism in CD4^+^ naïve T cells (28, 29). T cell senescence should be included in the assertion that cellular senescence is an emerging cardiovascular risk factor along with senescence of the endothelial and vascular smooth muscle cells (30, 31). We have reported that the absolute numbers of CD8^+^ naïve T cells decreased significantly with age in a nearly parallel pattern in HD patients aged 20–89 years (16). In the current study, we found that the levels of CD8^+^ naïve T cells dropped to an extremely low level in HD patients older than 65 years, which could explain why we did not find a significant correlation between CD8^+^ naïve T cells and infection in these patients. In the middle-aged patients, a decreased CD8^+^ naïve T cell count was significantly related to a higher risk of infection episodes. This could be attributed to a decreased T cell receptor diversity in naïve T cells, which are not only vital for a primary T cell response but continue to be a resource for T cell responses to antigens previously encountered. On the contrary, chronic immune stimulation, such as that by CMV infection, can also lead to the clonal expansion of the T cell population, which can severely compromise repertoire diversity. Recent studies indicated that ESRD patients present reduced T cell receptor diversity with clonal expansion (32, 33), leading to a high incidence of infection in these patients.

Generated from naïve T cells, T_CM_ cells home to lymph nodes, lack potent effector functions, and mount rapid secondary responses upon re-exposure to antigens. T_EM_ cells migrate to peripheral tissues and display immediate effector function at the sites of inflammation. T_EMRA_ cells are usually considered to be at an advanced stage of differentiation and are promoted by homeostatic cytokines or low load but protracted antigen exposure (34, 35). T_EMRA_ cells share the same characteristics as senescent cells, such as possessing short telomeres, DNA damage foci, and a secretome of senescence-associated secretory phenotype (36). Consistent with the concept that senescent cells exert systemic detrimental effects, T_EMRA_ cells have been implicated in several chronic disease states, such as rheumatoid arthritis, acute coronary syndromes, as well as poor vaccine responses (37–39). In the current study, T_EMRA_ cells were correlated with proinflammatory cytokines, such as TNF-*α* and IL-6. It is hard to distinguish causality between inflammation and expanded T_EMRA_ cells. In the present study, a higher percentage of T_EM_ cells was associated with CVEs, and a higher percentage of CD8^+^ T_EMRA_ cells was associated with infection. However, after including naïve T cells in the model, the association between these cells and clinical events diminished, indicating that an increase in differentiated T cells might partly be due to the decrease in naïve T cells; this is partly explained by some epigenetic studies (40, 41).

Overall, T-cell senescence in HD patients is markedly evident, and the contraction of the naïve T cell pool may act as a major player in developing CVEs and infections in these patients. Mechanistic studies on T cell homeostasis are needed in these patients. The central theme emerging from our finding is to alleviate chronic inflammation and promote cellular quiescence. Modifying HD therapy seems to be a feasible way to ameliorate T-cell inflammation and improve immunity against pathogens using antioxidant electrolyzed-reduced water (42) and introducing hemodiafiltration (43). To the best of our knowledge, only one study has investigated these T cell parameters in healthy individuals for each decade, with T cell subsets defined by co-expression of CD95 and CD62L, and reported that an increased absolute number of CD8^+^ memory T cells (CD95^+^CD62L^−^) correlated with increased mortality (44). Few other studies have reported the relevance of T-cell senescence to morbidity in the aged population. One study conducted in 1,072 elderly individuals from a nursing home indicated that a decreased percentage of CD4^+^ naïve T cells and CD8^+^ T_EM_ cells was correlated with frailty (45). In a case-control study conducted in 122 women aged 65 and above, no significant correlation was observed in naïve nor memory T cells between cases and controls (46). However, these studies did not take absolute count of these T cell parameters into consideration, which could miss the vital date of T-cell senescence in aged individuals. Thus, studying T-cell senescence in patients with ESRD can help to shed light onto the alteration of immune function in the general aged population.

Our study had several limitations. First, it remains unclear whether inflammation is the cause or the consequence of T-cell senescence. Second, T-cell senescence can be assessed by several other markers, such as telomere length, recent thymic emigrants, CD57, and CD28. This study cannot exclude the impact of these unmeasured parameters. Of particular interest is the fact that CMV infection has a substantial impact on T-cell senescence. In the current study, nearly all patients were seropositive for CMV-IgG, and half of them had an extremely high CMV-IgG titer, which could lead to underestimation of the relevance of CMV infection and T-cell senescence in HD patients. Finally, this was a single-center study, which might potentially limit the statistical power and its external validity. Hence, further studies are needed in this area to gain a deeper understanding.

In conclusion, HD patients exhibited accelerated immunosenescence in the T lymphocyte compartment, and these changes were positively related to inflammation. A reduction of naïve T cells was shown to be a strong predictor of CVEs and infection episodes in these patients. Monitoring naïve T cells could be useful for the early identification of patients at a high risk of profound complications.

## Data Availability Statement

The raw data supporting the conclusions of this article will be made available by the authors, without undue reservation.

## Ethics Statement

The studies involving human participants were reviewed and approved by Ethical Committee, Zhongshan Hospital, Fudan University. The patients/participants provided their written informed consent to participate in this study.

## Author Contributions

FX analyzed the data and drafted the manuscript. BS and JZ made the diagnosis and designed the experiments. XD, XHC, and ZZ revised the manuscript. FX and XSC collected the data. All authors contributed to the article and approved the submitted version.

## Funding

This work was supported by Natural Science Foundation of China (No. 82000705), Shanghai Municipal Hospital Frontier Technology Project supported by Shanghai Shen Kang Hospital Development Center (No. SHDC12018127), and Shanghai “science and technology innovation plan” popular science project (No. 19DZ2321400).

## Conflict of Interest

The authors declare that the research was conducted in the absence of any commercial or financial relationships that could be construed as a potential conflict of interest.
