# Supplementary material for: Decreased Peripheral Naïve T Cell Number and Its Role in Predicting Cardiovascular and Infection Events in Hemodialysis Patients
Source: Front Immunol. 2021 Mar 17;12:644627. doi: 10.3389/fimmu.2021.644627 (PMC8009982; doi:10.3389/fimmu.2021.644627)
Supplement: Supplementary file 1 [file DataSheet_1.docx]

# Supplementary Material

**Figure S1. Flowchart of flow cytometry analysis to identify T cell subset**


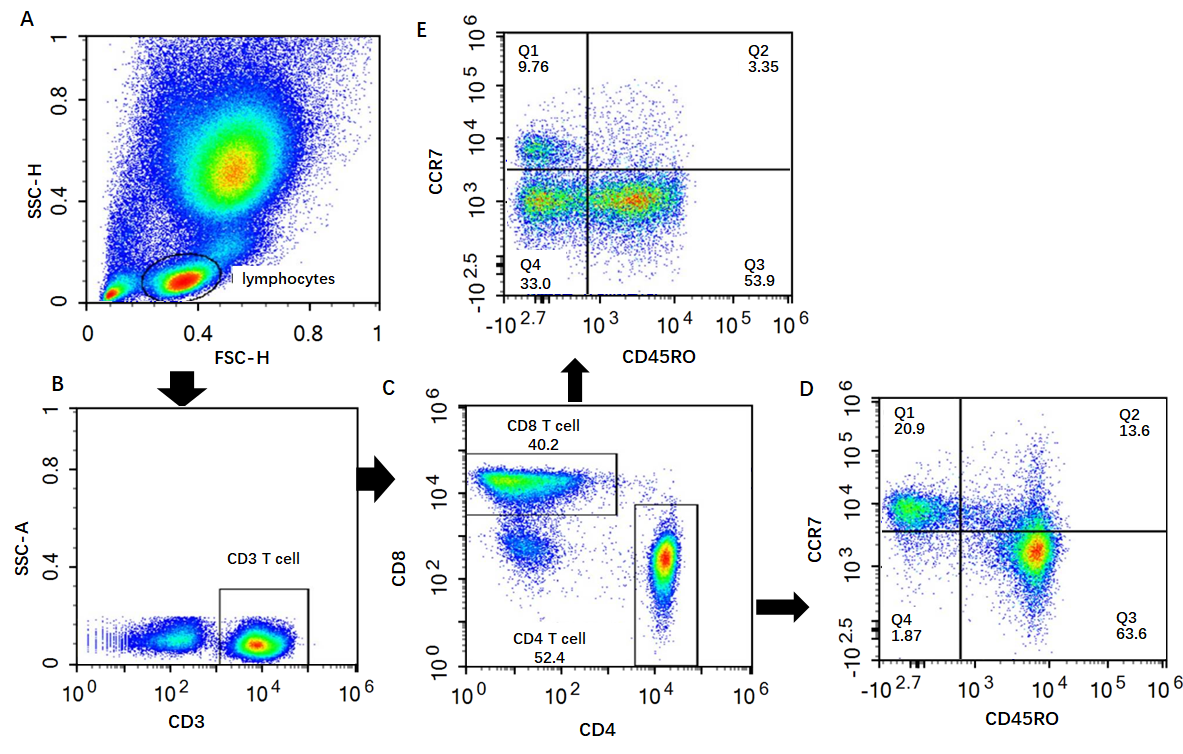


Figure S1. Flowchart of flow cytometry analysis to identify T cell subset. T cell subsets were defined by flow cytometry: Naïve T cells as CCR7+ and CD45RO-; central memory T cells as CD45RO+ and CCR7+; effector memory T cells as CD45RO+ and CCR7-, and EMRA T cells as CD45RO- and CCR7-.

**Figure S2.** **CD4^+^ T_Naïve_, CD4^+^ T_EM_ and CD8^+^T_EM_ were associated with CVE in hemodialysis patients**


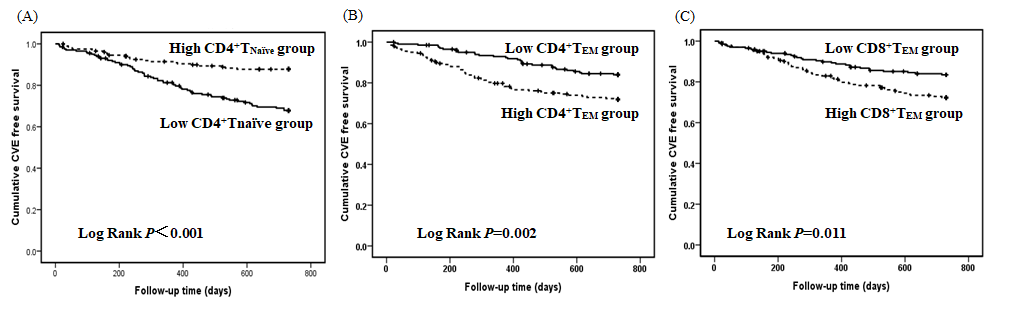


FigureS2. CD4^+^ T_Naïve_, CD4^+^ T_EM_ and CD8^+^T_EM_ were associated with CVE in hemodialysis patients

Median value of each T cell parameter was used in analyzing the correlation between CVEs. (A)Patients with lower count of CD4^+^ T_Naïve_ (＜137 cells/μl) had a significantly increased risk of CVE compared with those with higher count of CD4^+^ T_Naïve_ (≥137 cells/μl, *p*<0.001); (B) Patients with higher percentage of CD4^+^ T_EM_ (≥33.2%) had a significantly increased risk of CVE compared with those with lower percentage of CD4^+^ T_EM_ (＜33.2%, *p*=0.002); (C) Patients with higher percentage of CD8^+^ T_EM_ (≥22.0%) had a significantly increased risk of CVE compared with those with lower percentage of CD8^+^ T_EM_ (＜22.0%, *p*=0.011).

**Figure S3.** **CD8^+^ T_Naïve_ and CD8^+^ T_EMRA_ were associated with infection in hemodialysis patients**


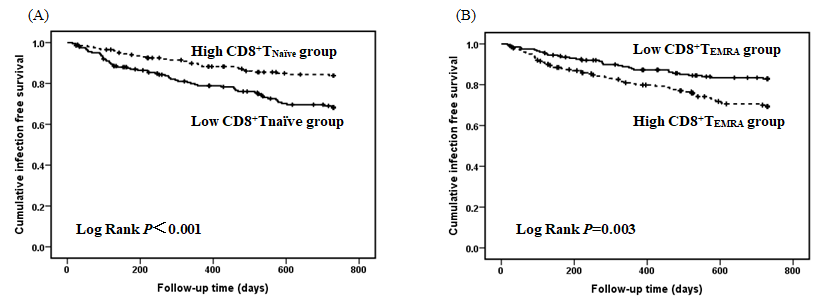


Figure S3. CD8^+^ T_Naïve_ and CD8^+^ T_EMRA_ were associated with infection in hemodialysis patients

Median value of each T cell parameter was used in analyzing the correlation between CVEs. (A) Patients with lower count of CD8^+^ T_Naïve_ (＜46 cells/μl) had a significantly increased risk of infection compared with those with higher count of CD8^+^ T_Naïve_ (≥46 cells/μl, *p*<0.001); (B) Patients with higher percentage of CD8^+^ T_EMRA_ (≥50.3%) had a significantly increased risk of infection compared with those with lower percentage of CD8^+^ T_EMRA_ (＜50.3%, *p*=0.003)
